# How does curvature affect the free-energy barrier of stalk formation? Small vesicles *vs* apposing, planar membranes

**DOI:** 10.1007/s00249-020-01494-1

**Published:** 2021-02-06

**Authors:** Y. G. Smirnova, M. Müller

**Affiliations:** Institute for Theoretical Physics, Georg-August University, 37077 Göttingen, Germany

**Keywords:** Vesicle, Membrane fusion, Simulation, Free-energy barrier

## Abstract

Using molecular simulations of POPC lipids in conjunction with the calculation of the Minimum Free-Energy Path (MFEP), we study the effect of strong membrane curvature on the formation of the first fusion intermediate—the stalk between a vesicle and its periodic image. We find that the thermodynamic stability of this hourglass-shaped, hydrophobic connection between two vesicles is largely increased by the strong curvature of small vesicles, whereas the intrinsic barrier to form a stalk, i.e., associated with dimple formation and lipid tails protrusions, is similar to the case of two, apposing, planar membranes. A significant reduction of the barrier of stalk formation, however, stems from the lower dehydration free energy that is required to bring highly curved vesicle into a distance, at which stalk formation may occur, compared to the case of apposing, planar membranes.

## Introduction

Changes of membrane topology such as pore formation, fusion, and fission are essential processes in the course of membrane remodeling (Bassereau et al. 2018), involved *inter alia* in cellular and subcellular trafficking (Chernomordik and Kozlov 2003; Jahn et al. 2003; Mattila et al. 2015), synaptic release of neurotransmitters (Südhof 2004; Rizzoli and Betz 2005; Zhou et al. 2013), viral infection (Chernomordik et al. 1998; Harrison 2008; Boonstra et al. 2018), and fertilization. These prototypical shape transformations are regulated by membrane-protein interactions, the local composition of the membrane, its local tension and dehydration, and its curvature. The dissection of the interplay of these different determinants is a challenge because the distinct factors often cannot be independently varied, and they may affect different stages of the change of membrane topology in different ways. Therefore, the influence of these factors on the qualitative mechanism of topology-altering membrane transformations and the quantitative free-energy barriers along the transformation path are only incompletely understood. Molecular simulation and modeling can contribute to our understanding by systematically varying the individual determinants in well-defined model systems, providing simultaneous information about the structure and geometry, and the free energy along the transformation path (Fuhrmans et al. 2015).

The classical perspective of membrane fusion divides the process into different stages (Chernomordik and Kozlov 2003). The zeroth stage—apposition—consists in bringing the membranes to be fused into close apposition. Proteins assert that the required membranes establish contact (recognition) and provide the free energy to overcome the hydration repulsion or alternate repulsion forces (*e.g.*, Helfrich repulsion or electrostatic forces) between membrane patches. The apposition also imparts a lateral tension onto the membranes. Importantly, molecular simulations of the coarse-grained MARTINI model (Marrink et al. 2007) suggest that the work, required to bring two membranes into apposition, significantly depends on the lipid species (Smirnova et al. 2019). Comparing lipid membranes comprised of phosphatidyl choline (POPC) or phosphatidylethanolamine (POPE) lipids, we found that the free-energy cost of establishing contact in the zeroth stage is smaller for POPE membranes that are characterized by a smaller headgroup and concomitantly more negative spontaneous curvature of a monolayer.

The first stage of fusion comprises the formation of the stalk – an hourglass-shaped hydrophobic connections between the apposing membrane. The excess free energy, $\varDelta F_{\mathrm{stalk}}$, of this first fusion intermediate with respect to the free energy of the apposing membranes, as well as the saddle-point and the associate free-energy barrier, $\varDelta F_{\mathrm{b}}$, which has to be overcome along the transformation path, have attracted abiding interest (Chernomordick et al. 1995; Kozlovsky et al. 2002; Jahn and Grubmüller 2002; Jahn et al. 2003). In our previous work, we have investigated the formation of this first fusion intermediate, connecting two planar membranes, by varying the intermembrane distance and spontaneous curvature of the lipid monolayers (using POPC and POPE lipids) (Smirnova et al. 2019). Using molecular simulations of the coarse-grained MARTINI model (Marrink et al. 2007), we obtained the thermodynamically reversible transition path between two apposing, planar bilayers and the stalk. We observed that the intrinsic free-energy barrier to form a stalk between apposing, planar membranes only weakly depends on the initial intermembrane distance, $d_\mathrm{w}$, between the membranes and the spontaneous curvature of the lipid monolayers. The stability of the metastable stalk intermediate, however, is largely defined by the intermembrane distance and, at distances of less than about 1 nm, the excess free energy, $\varDelta F_{\mathrm{stalk}}$, becomes negative. In this case, the thermal-equilibrium structure comprises a finite areal density of stalks that condense into a lattice or a rhombohedral structure formed between multilamellar stacks (Yang and Huang 2002; Aeffner et al. 2012). It speaks to the universality of amphiphilic self-assembly (Müller et al. 2003, 2006) that such structures have been first observed in diblock copolymer melts (Hajduk et al. 1997). Fusion proteins influence this first stage by (1) dictating the local membrane geometry at the site of stalk formation and (2) the interactions between the protein’s membrane anchors and the intermediate structures.

The second stage of fusion refers to the transition from the metastable stalk to a fusion pore. Several mechanisms have been observed involving the radially symmetric expansion of the stalk to a hemifusion diaphragm and its subsequent rupture (Kozlov and Markin 1983; Chernomordick et al. 1985) or the formation of a stalk-pore complex (Müller et al. 2003; Katsov et al. 2006) or stalk-peptide complex (Risselada et al. 2012).

In the present work, we focus on the role of membrane curvature on the fusion process. The role of membrane curvature is biologically relevant because e.g., synaptic release involves fast, SNARE-mediated fusion of neurotransmitter-containing vesicles with radii as small as 17–22 nm (Südhof 2004). Such small radii, which exceed the membrane thickness only by a factor of 4–6, alter the intrinsic membrane properties such as e.g., breaking the symmetry between the leaflets and resulting in a thinning of the bilayer. Membrane curvature is expected to affect all stages of the fusion process: (0) The free energy expended to bring the membrane into apposition qualitatively depends on the large-scale geometry of the membrane (Derjaguin 1934). (1) The excess free energy, $\varDelta F_{\mathrm{stalk}}$, as well as the barrier of stalk formation involve local nonlamellar intermediates, i.e., dimples and stalk, that connect to the large-scale membrane geometry. The geometric “fit” depends on the large-scale curvature at the fusion site. The large-scale geometry, in turn, affects the properties of the apposing *cis* monolayers, such as e.g., the areal density of headgroups in the apposing *cis* leaflets. (2) The large-scale geometry dictates the shape of the contact zone and may influence the second stage—opening of a fusion pore—by, e.g., facilitating the radial expansion of a stalk to an extended hemifusion diaphragm or directing the linear elongation of the stalk at the edge of the contact zone.

In the following, we focus on the role of membrane curvature on the first stage of fusion—the transition from apposing membranes to the stalk. Using self-consistent field theory (SCFT) calculations of mixed amphiphilic bilayers, Lee and Schick observed that the free-energy barrier to form a metastable stalk in vesicle-vesicle and vesicle-planar membrane fusion is hardly affected by the radius of the vesicle (Lee and Schick 2008). They also predict that the free-energy barrier in the second stage—from stalk to fusion pore—is reduced by membrane curvature, making the first stage rate-determining. Shinoda and coworkers, in turn, used molecular dynamics simulations of a coarse-grained model of lipid membranes in conjunction with a guiding potential to investigate the dependence of the free-energy profile of the fusion process on membrane curvature (Kawamoto et al. 2015). They observed that membrane curvature reduces the barrier to stalk formation, and results in a slightly wider stalk compared to a hydrophobic bridge between apposing, planar membranes. The vesicle’s curvature tends to stabilize the metastable stalk and, in agreement with the study of Lee and Schick (2008), gives rise to a more significant reduction of the barrier associated with the second transformation from stalk to fusion pore.

Here we extend our work on the thermodynamically reversible transition path (Smirnova et al. 2019) to stalk formation of highly curved POPC vesicles. Our manuscript is arranged as follows: in the next section—model and techniques—we provide details about the coarse-grained simulation model and free-energy techniques. The following section discusses our results. The manuscript closes with conclusions and a brief outlook.

## Model and technique

### Molecular model and simulation protocol

Coarse-grained simulations of a small POPC vesicle were performed using the GROMACS simulation package (Hess et al. 2008) and the MARTINI force field (version 2.0) with non-polar water (Marrink et al. 2007). The vesicle was formed by spontaneous aggregation, following the protocol from Risselada et al. (2008, 2014). It contains 1447 lipids in the outer leaflet and 770 lipids in the inner leaflet, with the total number of 97,217 solvent beads. The simulation box was $26.31\times 26.83\times 20.74\,\hbox {nm}^3$, and the vesicle outer radius was $R_\mathrm{v}=9.2\,\hbox {nm}$.

After initial equilibration in the NPT ensemble at temperature $T=300\,$K, we simulated the vesicle in the NVT ensemble to study the formation of a stalk between the vesicle and its periodic image (Risselada et al. 2014). This is equivalent to the fusion of two independent vesicles if the area of the vesicle is large compared to the area involved in fusion (or the excess number of particles in the stalk). This setup significantly reduces the simulation time due to the smaller system size compared to a system comprised of two vesicles but it also introduced some restrictions. For instance, the box dimensions are fixed in the NVT ensemble and do not adapt in the course of the change of membrane topology. Therefore, the membrane tension may slightly vary along the transformation path. Previous simulations, however, indicate that this effect on the excess free energy of the stalk is only of the order of the thermal energy unit, $k_{\mathrm{B}}T$ (Norizoe et al. 2010; Smirnova et al. 2019). We shall verify this expectation below, by showing that the stalk structure is very similar in the NVT and NPT ensembles. Additional details of the setup of the small system comprised of two, apposing, planar membranes are provided in Smirnova et al. (2019). Since the excess of lipid molecules in the stalk structure is small (Norizoe et al. 2010; Daoulas and Müller 2013) finite-size effects are presumably not significant. The intermembrane distance, $d_\mathrm{w}$, between the two “halves” of the vesicle in the starting configuration is about the minimal distance that vesicles can spontaneously attain due to the strong hydration repulsion on short distances. Note that the MARTINI model provides a rather quantitative description of the intermembrane repulsion despite the coarse-grained representation of water and lipid headgroups (Smirnova et al. 2013).

### Order parameter, $m(\mathbf{c})$, and free-energy function, $F(\{m(\mathbf{c})\})$

To quantify the free-energy profile along the transformation from two apposing, highly curved vesicles to a stalk, we compute the Minimum Free Energy Path (MFEP) and compare this result with the MFEP from two apposing planar membranes and a stalk (Smirnova et al. 2019). We characterize the configuration of the system by a spatially varying, collective order-parameter field, $m(\mathbf{r})$, and assume that all other degrees of freedom are in equilibrium along the transformation path. In accord with our previous studies (Smirnova and Müller 2015; Smirnova et al. 2019), we choose the density of hydrophobic lipid particles as order parameter because the system is nearly incompressible. This choice of the order parameter implies that the individual molecular conformations at each point along with the transformation path sample all available configurations given the constraint on the collective density of hydrophobic lipid particles, i.e., the hydrophobic density is the only slow variable.

In the computations, we discretize three-dimensional space by a collocation grid with 90 or 20 grid points along each Cartesian direction for the vesicles or apposing, planar membranes, respectively. This choice asserts that a few particles contribute to the density at a grid point. A grid cell, $\mathbf{c}$, corresponds to a volume of $\varDelta V=\varDelta L_x\varDelta L_y\varDelta L_z= 0.02\,\hbox {nm}^3$ for the vesicle system or $0.09 \,\hbox {nm}^3$ for the planar-membrane system. Summing over all hydrophobic particles with positions, ${\hat{\mathbf{r}}}_{i_h}$, we map a microscopic particle configuration in three-dimensional space onto an order-parameter configuration on the collocation grid

1 $$\begin{aligned} {{\hat{m}}}(\mathbf{c})= \frac{1}{\varDelta V}\sum _{i_h} \Pi (\mathbf{c},{{\hat{\mathbf{r}}}}_{i_h}) \end{aligned}$$

For molecular dynamic simulation we require that the mapping between position and order parameter be continuous (Smirnova and Müller 2015) and use a linear assignment

2 $$\begin{aligned}&\Pi (\mathbf{c},\mathbf{r}) = \prod _{\alpha \in \{x,y,z\}} \pi _\alpha (|\mathbf{r}_{\alpha }-\mathbf{c}_{\alpha }|) \nonumber \\&\quad \text{ with } \quad \pi _\alpha (d) = \left\{ \begin{array}{ll} 1-\frac{|d|}{\varDelta L_\alpha } &{} \quad \text{ for } \quad |d| \le \varDelta L_\alpha \\ 0 &{} \quad \text{ otherwise } \end{array}\right. \end{aligned}$$

where $\mathbf{c}_\alpha$ denotes the Cartesian coordinate of the center of the grid cell, $\mathbf{c}$. Further details of the implementation can be found in Smirnova and Müller (2015).

A thermodynamic state is defined by all microscopic configurations that give rise to the same set of hydrophobic density, $\{m(\mathbf{c})\}$, on the collocation grid. Thus, $m(\mathbf{c})$ completely specifies the thermodynamic, nonequilibrium state. We associate the free-energy function, $F(\{m(\mathbf{c})\})$, to each thermodynamic state, $m(\mathbf{c})$, according to

3 $$\begin{aligned}
F(\{m({\bf c})\}) \equiv - k_{\rm B}T \ln \int {\cal D}[\{\hat{\bf r}_{i}\}] \; \exp\left(-\frac{{\cal V}(\{\hat{\bf r}_{i}\})}{k_{\rm B}T} \right) \; \prod_{\bf c}\delta\left(m({\bf c}) - \hat m({\bf c}) \right) 
\end{aligned}$$

The integral sums over all microscopic particle coordinates, accounting for the indistinguishability of lipids and normalized by the thermal de-Broglie wavelength. ${{{\mathcal {V}}}}(\{{{\hat{\mathbf{r}}}}_{i}\})$ denotes the potential energy of the MARTINI force field (Marrink et al. 2007). Note that $F(\{m(\mathbf{c})\})$ is the discretized version of a free-energy functional. It is a high-dimensional function that depends on $N_c=90^3$ or $20^3$ variables for the vesicle or planar-membrane system, respectively.

### Minimum free-energy path (MFEP) and string method

In the following, we study the transformation from two apposing vesicles (or rather one vesicle with periodic boundary conditions) to a state where the vesicles are connected by a stalk. The starting and the ending state—vesicle and stalk—correspond to local minima of $F(\{m(\mathbf{c})\})$.

We quantify the transformation by a path, $m_s(\mathbf{c})$, in the $N_c$-dimensional order-parameter space, where $0\le s \le 1$ denotes the contour parameter along the path. *s* quantifies the progression of the transformation: $s=0$ corresponds to the starting state—vesicle—and $s=1$ represents the ending state—stalk. The Minimum Free-Energy Path (MFEP) is the most likely transition path between the starting and ending states, and it is defined by the condition that the thermodynamic driving force—the chemical potential $\mu _s(\mathbf{c})=\frac{\partial F}{\varDelta V \partial m_s(\mathbf{c})}$ – in the direction perpendicular to the path vanishes, i.e.,

4 $$\begin{aligned} \varDelta V \left. \mu _{s}(\mathbf{c})\right| _{\perp }\equiv & {} \left. \frac{\partial F}{\partial m_{s}(\mathbf{c})}\right| _{\perp } \nonumber \\= & {} \frac{\partial F}{\partial m_{s}(\mathbf{c})} - \frac{\mathrm{d}m_{s}(\mathbf{c})}{\mathrm{d}s} \frac{ \sum _\mathbf{c} \frac{\partial F}{\partial m_{s}(\mathbf{c})} \frac{\mathrm{d}m_{s}(\mathbf{c})}{\mathrm{d}s} }{ \sum _\mathbf{c} \left( \frac{\mathrm{d}m_{s}(\mathbf{c})}{\mathrm{d}s} \right) ^{2} } \mathop = \limits^{!} 0. \end{aligned}$$

for all $0\le s \le 1$ and grid points, $\mathbf{c}$.

To compute the MFEP of stalk formation (Müller et al. 2012; Ryham et al. 2016; Smirnova et al. 2019; Han et al. 2020), we employed the string method (Maragliano et al. 2006; E et al. 2007). We discretize the transformation path into $n=24$ replicas for the vesicle and $n=19$ for the apposing, planar membranes. The string algorithm iterates a cycle of two steps: (1) minimize the free energy of each replica via Allen-Cahn dynamics (Halperin and Hohenberg 1977), $\varDelta m_{s_i}(\mathbf{c}) \sim - \frac{\partial F}{\partial m_{s_i}(\mathbf{c})}$ for $i=1,2,\cdots ,n$, and (2) parameterize the string of morphologies $m_{s_i}(\mathbf{c})$ at each point $\mathbf{c}$ by a cubic spline in the variable *s* and redistribute the replicas uniformly along the string. Here the distance, $\varDelta _i$ between two replica, $s_{i+1}$ and $s_i$ is given by (Müller et al. 2012) $\varDelta _i^2 = {{{\mathcal {N}}}}\sum _\mathbf{c} \left[ m_{s_{i+1}}(\mathbf{c})-m_{s_i}(\mathbf{c}) \right] ^2$ where the normalization constant is chosen such that $\sum _i \varDelta _i=1$.

The initial order parameter at the starting and ending points has been obtained by averaging the hydrophobic density in the metastable states—vesicle, $s_{i=1}=0$, and stalk, $s_{i=n}=1$. The order parameter of intermediate replica has been constructed by pointwise, linear interpolation of $m_s(\mathbf{c})$ between $s=0$ and $s=1$ for the vesicle system. For planar membranes, in turn, we used configurations of previous work by Smirnova et al. (2010). After about 200–400 iteration cycles the string algorithm converges to the MFEP. The MFEP provides a thermodynamically reversible path between the starting and ending configuration that passes through the saddle-point(s) on the free-energy landscape, $F(\{m(\mathbf{c})\})$ – given the choice of the order parameter, it is the optimal reaction coordinate.

The free-energy function, $F(\{m(\mathbf{c})\})$, that describes the molecular system is unknown. Fortunately, the string algorithm only requires its derivative—the chemical potential. The latter quantity can be obtained by field-theoretic umbrella sampling (Müller 2011; Müller et al. 2012; Smirnova and Müller 2015; Smirnova et al. 2015). To this end, we restrain fluctuations of the microscopic order parameter, ${{\hat{m}}}(\mathbf{c})$ at grid point $\mathbf{c}$, from the given collective value, $m(\mathbf{c})$, by an umbrella potential

5 $$\begin{aligned} \frac{\varDelta H_{\mathrm{fup}}(\{\mathbf{r}\})}{k_{\mathrm{B}}T} = \frac{\lambda }{2} \varDelta V \sum _\mathbf{c} \Big ( m(\mathbf{c}) - {{\hat{m}}}(\mathbf{c})\Big )^{2} \end{aligned}$$

The strength of the field-theoretic umbrella potential is set to $\lambda /\varDelta V=50\,\hbox {kJ/mol}$ (Gromacs units). In the limit of large $\lambda$, the spatially varying chemical potential that corresponds to a nonequilibrium order parameter, $m_s(\mathbf{c})$, is given by

6 $$\begin{aligned} \mu _s(\mathbf{c}) = \frac{\partial F}{\varDelta V\partial m_{s}(\mathbf{c})} \approx k_{\mathrm{B}}T \lambda \left[ m_s(\mathbf{c}) - \left\langle {{\hat{m}}}_s(\mathbf{c})\right\rangle _{\mathrm{fup}} \right] \end{aligned}$$

This estimate is accurate up to order $\lambda ^{-1}$, though higher-order schemes are available (Sun and Müller 2018). The average, $\langle {{\hat{m}}}_s(\mathbf{c})\rangle _{\mathrm{fup}}$, over the microscopic hydrophobic density in the presence of $\varDelta H_{\mathrm{fup}}$ is extended over 10 ns.

Since the MFEP is a thermodynamically reversible path in the high dimensional space, $m_s(\mathbf{c})$, we use thermodynamic integration to obtain the free-energy profile, $\varDelta F_s$, along the path. We refer all free-energy differences to the free energy of the starting state – vesicle or apposing, planar membranes, respectively.

7 $$\begin{aligned} \varDelta F_s = \int _0^s\mathrm{d}s'\; \varDelta V\sum _\mathbf{c}\; m_{s'}(\mathbf{c}) \mu _{s'}(\mathbf{c}), \end{aligned}$$

where we use pointwise cubic splines to interpolate between the replica. The maximum, $\varDelta F_{\mathrm{b}}=\max _s\varDelta F_{s}$ defines the barrier to stalk formation, whereas $\varDelta F_{\mathrm{stalk}}=\varDelta F_{s=1}$ quantifies the excess free energy of the stalk.

Further details of the application of the string method and the field-theoretic umbrella potential for simulation of membranes can be found in Smirnova and Müller (2015) and Smirnova et al. (2019). Here we briefly summarize the simulation protocol: We used the MARTINI force field for POPC lipids and non-polar water version 2.0 (Marrink et al. 2007). The path was described by 24 system replica for the vesicle system, and 19 system replica for the planar membranes. Each replica was characterized by the order parameter—hydrophobic lipid density (only lipid tails beads)—which was calculated using Eq. 1 on the lattice with unit size, $\varDelta V=0.3\times 0.3\times 0.2\,\hbox {nm}^3$, for the vesicle system and, $\varDelta V=0.3\times 0.3\times 0.8\,\hbox {nm}^3$, for the planar membranes with $90^3$ and $20^3$ grid points, respectively. A field-theoretic umbrella potential was used to restrain the system configuration to a given order parameter. The force constant of this field-theoretic umbrella potential per unit cell volume was set to $\lambda /\varDelta V=50\,\hbox {kJ/mol}$. The external force generated due to this potential was added to the force calculation at each MD step for lipid tails beads. Each simulation run for the order parameter update was 10 ns. We used Allen-Cahn dynamics to generate a new order parameter, $m_{\mathrm{new}}(\mathbf{c})=m_{\mathrm{old}}(\mathbf{c})-\epsilon \mu _s(\mathbf{c})$, with the time step $\epsilon \lambda /\varDelta V=0.03$. For each update, the free energy profile along the path was integrated according to Eq. 7. The convergence of the minimum free energy path was typically reached in about 200 updates for the small system and 400 updates for the large vesicle system.

## Results and discussion

### Zeroth stage: apposition

**Fig. 1:**
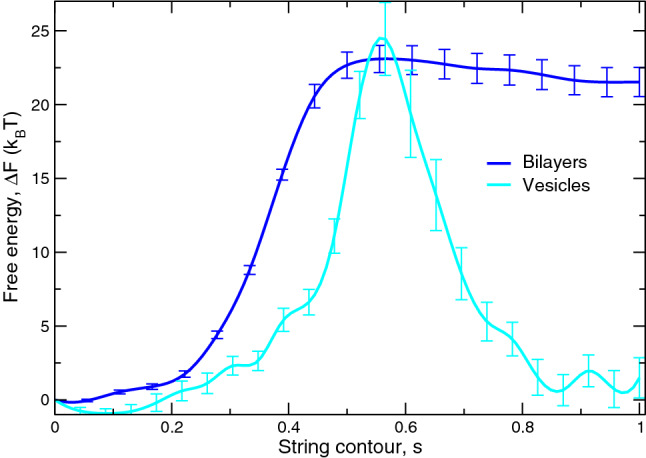
Minimum Free Energy Path (MFEP) of stalk formation between two apposing, planar bilayers (blue curve) and a small vesicle and its periodic image (cyan curve). Standard errors are shown with bars. $s=0$ corresponds to closely apposing, planar bilayers with an intermembrane distance of $d_\mathrm{w}=1.2\,\hbox {nm}$ in the starting state, whereas the vesicle and its periodic image initially are separated by the minimal distance, $d_\mathrm{w}=2.3\,\hbox {nm}$

In the zeroth stage of fusion, the membranes to be fused are brought into close apposition. The excess free energy, $\varDelta F_{\mathrm{stalk}}$, sensitively depends on the initial distance—quantified by the minimal thickness, $d_\mathrm{w}$, of the water layer—between the membranes. Smaller distances *i.e.*, higher dehydration, significantly increase the (meta)stability of the stalk (Aeffner et al. 2012; Smirnova et al. 2019). In accord with the experimentally measured intermembrane distances, $d_\mathrm{w}$, and membrane thicknesses (Aeffner et al. 2012), we define the membrane interface at equal densities of phosphate and glycerol units and calculate $d_\mathrm{w}$ *via* the density profiles across the membrane (Smirnova et al. 2013). For apposing, planar membranes the hydrophobic density at this position is $2.2\,\hbox {nm}^{-3}$. In the following, we choose the distance between the membranes in the starting state to be about 2.3 nm. At this distance, the excess free energy, $\varDelta F_{\mathrm{stalk}}$, between the vesicle and its periodic image approximately vanishes (see Fig. 1).

For distances smaller than 2 nm, the hydration repulsion between vesicles starts to give rise to an important contribution to the free energy, and vesicles cannot spontaneously approach each other (Smirnova et al. 2013). The excess free energy due to the hydration repulsion between two vesicles at close distances can be written according to Derjaguin approximation (Derjaguin 1934) for colloids (Russel et al. 1989)

8 $$\begin{aligned} \varDelta F_{\mathrm{dehydr}}(d_\mathrm{w})=\pi R_\mathrm{v}\lambda \varDelta f_{\text {flat}}(d_\mathrm{w}), \end{aligned}$$

Here, $R_\mathrm{v}$ denotes the radius of the outer interface of the vesicle, and we have assumed that the hydration repulsion varies exponentially with the distance, $d_\mathrm{w}$ (Smirnova et al. 2013). $\lambda =0.28\,\hbox {nm}$ is the decay length of the hydration repulsion for POPC lipids (Smirnova et al. 2013) and $\varDelta f_{\text {flat}}$ is the hydration-repulsion free energy per unit area of two, apposing, planar membranes.

The larger the vesicle’s curvature, $1/R_\mathrm{v}$, the smaller the hydration repulsion between the vesicles. The corresponding excess free energy, $\varDelta F_{\text {flat}}(d_\mathrm{w})= A f_{\text {flat}}(d_\mathrm{w})$ for two apposing planar membranes ($R_\mathrm{v}\rightarrow \infty$), is proportional to the contact area, *A*, and, therefore, it is not well defined.

At the intermembrane distance $d_\mathrm{w}=2.3\,\hbox {nm}$, the stalk that connects the vesicle and its periodic image has a vanishingly small excess free energy. At this intermembrane distance, however, we do not observe a metastable stalk between two apposing, planar membranes. The stalk structure quickly looses its (meta)stability with increasing intermembrane distance, $d_\mathrm{w}$, between planar membranes (Smirnova et al. 2019) and, for distances $d_\mathrm{w}>1.24\,\hbox {nm}$, it becomes absolutely unstable compared to the two, apposing, planar membranes. Therefore, here we choose the apposing, planar membrane system with largest intermembrane distance, where the stalk is observed to be a metastable state, $d_\mathrm{w}=1.2\,\hbox {nm}$, for the comparison.

Establishing such a short distance between apposing, planar membranes gives rise to a significant excess free-energy contribution from dehydration (Smirnova et al. 2013), which for POPC bilayers is about 1.2 $k_{\mathrm{B}}T/\hbox {nm}^2$, and for the patch size of $36 \,\hbox {nm}^2$, this approximately amounts to 43 $k_{\mathrm{B}}T$. For the vesicle case, in turn, the intermembrane distance of 2.3 nm can be spontaneously reached by the system. Even bringing two small vesicles with $R_\mathrm{v}=9.2\,\hbox {nm}$ to the same short distance, $d_\mathrm{w}=1.2\,\hbox {nm}$, we only have to use about $10 \, k_{\mathrm{B}}T$, according to Eq. 8.

Thus, the free energy expended in the zeroth stage of fusion—apposition—is very different for apposing, planar membranes and highly curved vesicles. This difference, however, is not considered in the following because it is proportional to the area of the apposing, planar membranes or to the concomitant divergent radius of the contact zone, according to Eq. 8.

### First stage: stalk formation

Figure 1 presents the MFEPs for stalk formation between a small vesicle and its periodic image and compares this result with the MFEP of stalk formation between apposing planar membranes. As discussed above, the minimal distance between the membranes in the starting state is $d_\mathrm{w}=2.3\,\hbox {nm}$ for the vesicle but only 1.2 nm for the two apposing, planar membranes. Comparing the free-energy profiles of stalk formation between apposing, planar membranes and vesicles at these different distances, we observe that the barrier for stalk formation, $\varDelta F_{\mathrm{b}}$, is comparable and amounts to $24\pm 3 k_{\mathrm{B}}T$. Previous work on stalk formation between apposing, planar membranes has demonstrated that $\varDelta F_{\mathrm{b}}$ is rather insensitive to the intermembrane distance, $d_\mathrm{w}$.

The excess free energy, $\varDelta F_{\mathrm{stalk}}$, of the stalk state, however, markedly differs. It is about $2.4\pm 1.4 \, k_{\mathrm{B}}T$ for vesicles but amounts to $21.5\pm 1 \, k_{\mathrm{B}}T$ in the case of two apposing, planar membranes. If we compared the excess free energy of the stalk state at comparable distances, the stalk between small vesicles would be significantly more stable (when we reduced $d_\mathrm{w}$ for the vesicle system) or the stalk structure would not even be metastable (when we increased $d_\mathrm{w}$ for the system of apposing, planar membranes).

**Fig. 2:**
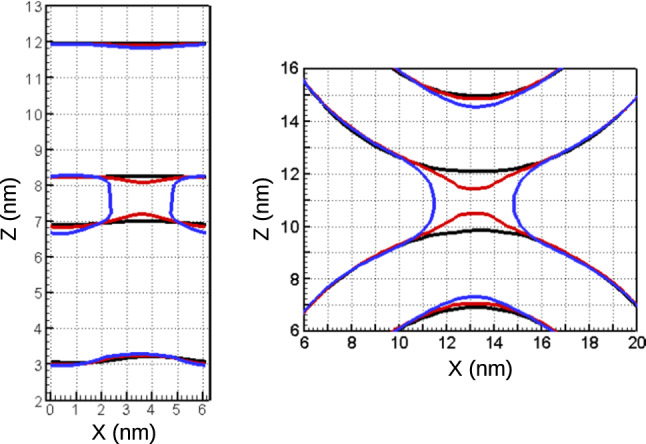
Contour lines on (*x*, *z*) plane, corresponding to the hydrophobic number density 2.2 $\hbox {nm}^{-3}$, for the structures along the MFEP for the planar bilayers, $s=0$ (black), $s=0.28$ (red) and $s=1$ (blue), and for the vesicles, $s=0.09$ (black), $s=0.53$ (red) and $s=1$ (blue) zoomed in the region of the stalk. The contour lines are shown for the slices corresponding to the center of the stalk, $y=3.8\,\hbox {nm}$, for the bilayers and, $y=13\,\hbox {nm}$, for the vesicle system

To illustrate the membrane shape along the transformation path, we depict in Fig. 2 the positions of the membrane interface at selected contour parameters, *s*, of the MFEPs for two apposing, planar membranes (left panel) and the vesicle (right).^1^ Note that we use a rather low threshold of the hydrophobic density to define the membrane interface; if we increased the threshold, the distance, $d_\mathrm{w}$, between the membranes would be slightly larger, the diameter of stalk structures would be slightly smaller, but the qualitative behavior would remain unaltered.

For the system of apposing, planar membranes, the starting state, $s=0$, corresponds to the intermembrane distance $d_\mathrm{w}=1.2\,\hbox {nm}$. This minimal distance slowly decreases along the transformation path, and the excess free energy rises in turn. At $s=0.22$ the intermembrane distance has decreased to $d_\mathrm{w}\approx 1\,\hbox {nm}$, corresponding to an excess free energy $\varDelta F_{s=0.22}=1.7\pm 0.2\, k_{\mathrm{B}}T$. At $s=0.28$, we observe an intermembrane distance $d_\mathrm{w}=0.85\,\hbox {nm}$ and the local membrane deformation—dimple—becomes more pronounced as the proximal, *cis* leaflets of both membranes approach each other, as depicted in Fig. 2. The bilayer structure, where the hydrophilic headgroups shield the hydrophobic tails from the solvent, however, remains largely intact. This dimple deformation yields $\varDelta F_{s=0.28}=4.4\pm 0.3 \, k_{\mathrm{B}}T$.

Farther along the transformation path, $s=0.33$, a very narrow hydrophobic connection between the apposing, planar membranes is formed. We denote this structure as pre-stalk. Its diameter, $d_{\mathrm{st}}$, only amounts to about 0.84 nm, and thereby it is comparable to the size of a hydrophobic MARTINI bead, 0.47 nm. In this initial hydrophobic bridge the hydrophobic lipid tails—either in form of protrusions or splayed lipid tails that straddle between the two apposing membranes—become exposed to the hydrophilic surroundings, resulting in a steep rise of the free-energy profile, $\varDelta F_\mathrm{s}$.

In some sense, this pre-stalk is the analog of a pre-pore (or a hydrophobic pore) in membrane poration (Abidor et al. 1979; Ting et al. 2018), where the topological change—here, the connection between the hydrophobic tail regions of the apposing, planar membranes, or a hydrophilic path through a membrane in case of pore formation—has occurred but the headgroups have not yet re-arranged.

Eventually, the barrier is reached at $s\approx 0.56$, and this goes along with the formation of a proper stalk, where headgroups shield the hourglass-shaped hydrophobic bridge from the surrounding solvent. The subsequent widening of the stalk slightly reduces the free energy until the metastable stalk state is reached at $s=1$.

The shape changes in the case of vesicles are qualitatively similar. First, the free-energy profile, $\varDelta F_\mathrm{s}$, also slowly increases between $s=0.09$ and 0.43 where the minimal distance decreases from $d_\mathrm{w}=2.3\,\hbox {nm}$ towards $d_\mathrm{w}=2.1\,\hbox {nm}$. The formation of a pre-stalk at around $s\approx 0.56$ is preceded by a dimple-like deformation of the apposing, *cis* leaflets. Since the initial distance between the vesicles is larger, $d_\mathrm{w}=2.3\,\hbox {nm}$, the dimple-like deformation at $s=0.53$ (see Fig. 2) is more pronounced than in the case of closely apposing, planar membranes, and gives rise to a steep increase of $\varDelta F_\mathrm{s}$.

The initial hydrophobic bridge—pre-stalk—at $s\approx 0.54$ only has a diameter of $d_{\mathrm{st}} \approx 1.4\,\hbox {nm}$. The saddle-point occurs at only slightly larger $s=0.57$. Thereafter the excess free energy significantly decreases as headgroups shield the hydrophobic bridges and the diameter of the stalk widens. The reduction of the excess free energy upon widening the hydrophobic connection after the barrier is significantly more pronounced for the vesicle system than for the system of apposing, planar membranes.

To provide an intuitive, physical description of the MFEP in the high-dimensional order-parameter space, it is useful to characterize the changes in morphology with respect to *s*. These changes approximately correlate with two simple, physical quantities (Müller et al. 2012; Smirnova et al. 2015): For small *s* the minimal distance, $d_\mathrm{w}$, is an appropriate reaction coordinate, whereas the diameter, $d_{\mathrm{st}}$ of the stalk describes the progress of the shape transformation at larger *s*. The switch between these reaction coordinates occurs in the pre-stalk region, i.e., before the barrier for the system of two apposing, planar membranes or only slightly before in the case of stalk formation between vesicles.

**Fig. 3:**
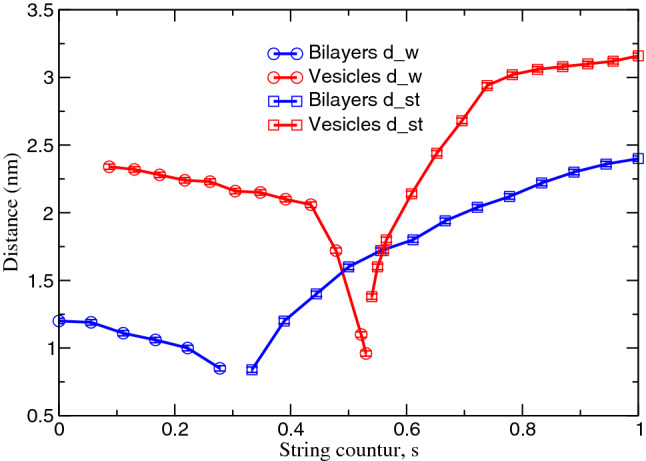
The intermembrane distance, $d_\mathrm{w}$, and the stalk diameter, $d_{\mathrm{st}}$, along the MFEP of the stalk formation for the system of apposing, planar membranes (blue) and small vesicles (red). The distances were determined using the hydrophobic number density $2.2\,\hbox {nm}^{-3}$

The changes of these two physical descriptions of the MFEP—the intermembrane distance, $d_\mathrm{w}$, and the diameter, $d_{\mathrm{st}}$, of the pre-stalk or stalk—along the two MFEPs are presented in Fig. 3.

For the case of apposing, planar membranes, the initial hydrophobic connection—pre-stalk—has a diameter of about 0.84 nm, whereas the saddle-point of the free-energy landscape corresponds to a stalk with diameter, $d_{\mathrm{st}} \approx 1.7\,\hbox {nm}$. The metastable stalk structure, $s=1$, on the MFEP has the diameter 2.4 nm.

In the case of stalk formation between small vesicles, in turn, the initial diameter of the hydrophobic bridge and its value at the saddle-point are rather similar, $d_{\mathrm{st}} \approx 1.4\,\hbox {nm}$ and $d_{\mathrm{st}} \approx 1.8$, respectively, and upon progression of the shape transformation, the stalk diameter grows and reaches a plateau, corresponding to about $d_{\mathrm{st}}=3.2\,\hbox {nm}$. Thus, in qualitative accord with previous simulations by Shinoda and coworkers (Kawamoto et al. 2015), we observe that the metastable stalk between two vesicles is wider than that between two apposing, planar membranes although the initial distance between the apposing, planar membranes is significantly smaller than the minimal initial distance between the vesicles.

**Fig. 4:**
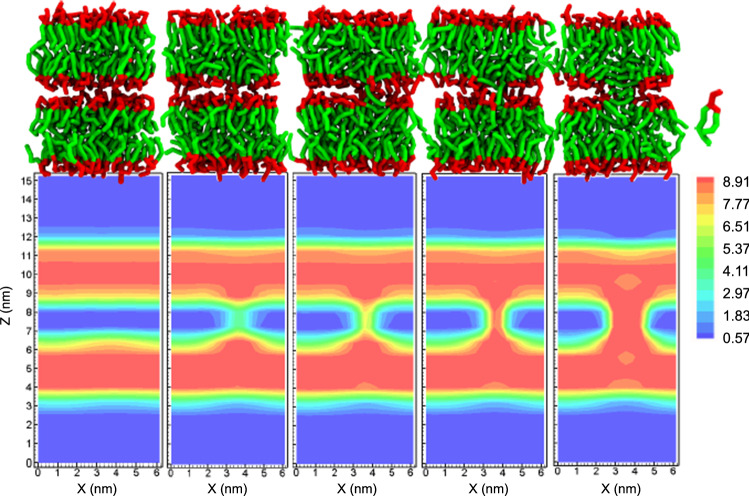
Snapshots of apposing, planar membranes along the transformation path (top panel) and the corresponding averaged hydrophobic density slices, $y=3.8\,\hbox {nm}$, (bottom panel) for $s=0$, $s=0.39$, $s=0.44$, $s=0.5$, $s=1.0$. The color scale depicts the hydrophobic number density in $\hbox {nm}^{-3}$. POPC lipids are illustrated by hydrophobic beads in green color and hydrophilic beads in red color. Solvent beads are not depicted in this representation

**Fig. 5:**
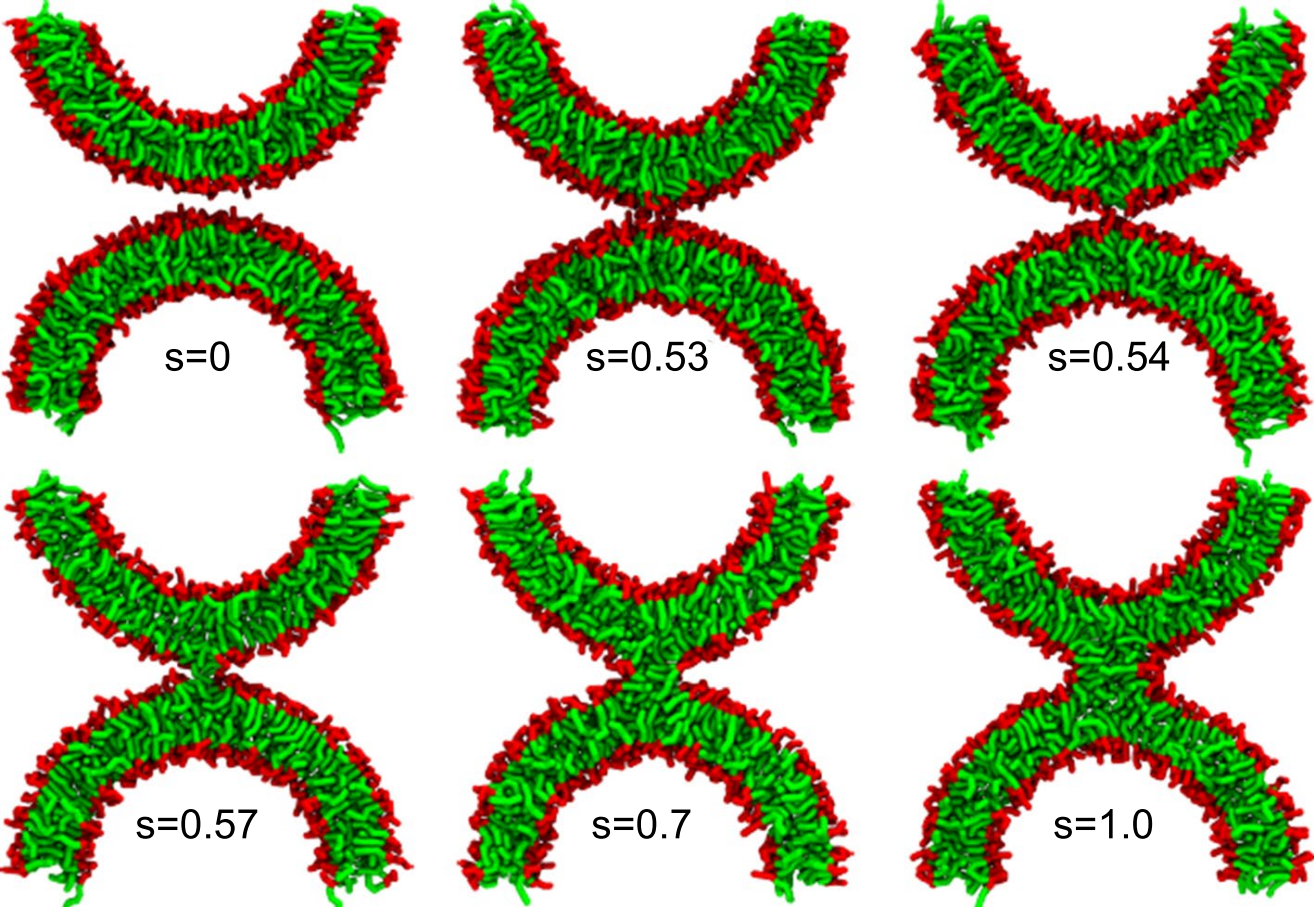
Snapshots of a vesicle, forming a stalk across the periodic boundary conditions, at various contour positions along the MFEP, $s=0.09$, $s=0.53$, $s=0.54$, $s=0.57$, $s=0.7$ and $s=1.0$. Hydrophobic beads of the POPC lipids are shown in green color and hydrophilic beads in red. Solvent beads are omitted in this representation

**Fig. 6:**
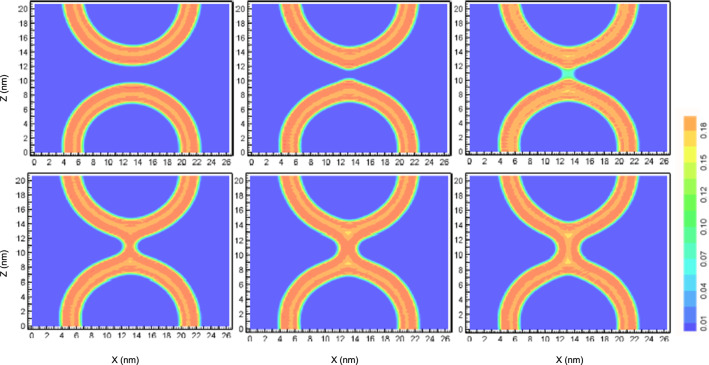
Contour slices, $y=13\,\hbox {nm}$, of the averaged hydrophobic density for $s=0.09$, $s=0.53$, $s=0.54$, $s=0.57$, $s=0.7$ and $s=1.0$. The color scale depicts the hydrophobic number density in $\hbox {nm}^{-3}$

Instantaneous snapshots of the MARTINI-model simulations with the field-theoretic umbrella potential and the corresponding hydrophobic membrane densities are shown in Fig. 4 along the MFEP of stalk formation between apposing, planar membranes. The corresponding data for the vesicle system are compiled in Figs. 5 and 6.

The snapshots of the molecular simulations, Fig. 4 (top) and Fig. 5, assert that the initial hydrophobic bridge between the membranes typically consists of solvent-exposed lipid tails that are splayed and make contact to both *cis* leaflets (Smirnova et al. 2010; Mirijanian et al. 2010) or simply tail protrusions, both in the case of apposing, planar membranes as well as vesicles. Given the low threshold on the hydrophobic density that we used to identify the membrane interface, these strongly fluctuating structures are identified as initial stalks, cf. snapshots and order parameter for $s=0.39$ in the case of apposing, planar membranes and $s=0.54$ in the case of vesicles.

Additionally, we note that in the metastable stalk, $s=1$, the hydrophobic density is decreased at the top and bottom hydrophobic interstices of the stalk, where the membrane leaflets with different curvatures contact each other. This small reduction of the hydrophobic density in this nonlamellar region signals local packing frustration (Kozlovsky and Kozlov 2002). This effect appears to be somewhat more pronounced in the vesicle case than for the apposing, planar membranes.

**Fig. 7:**
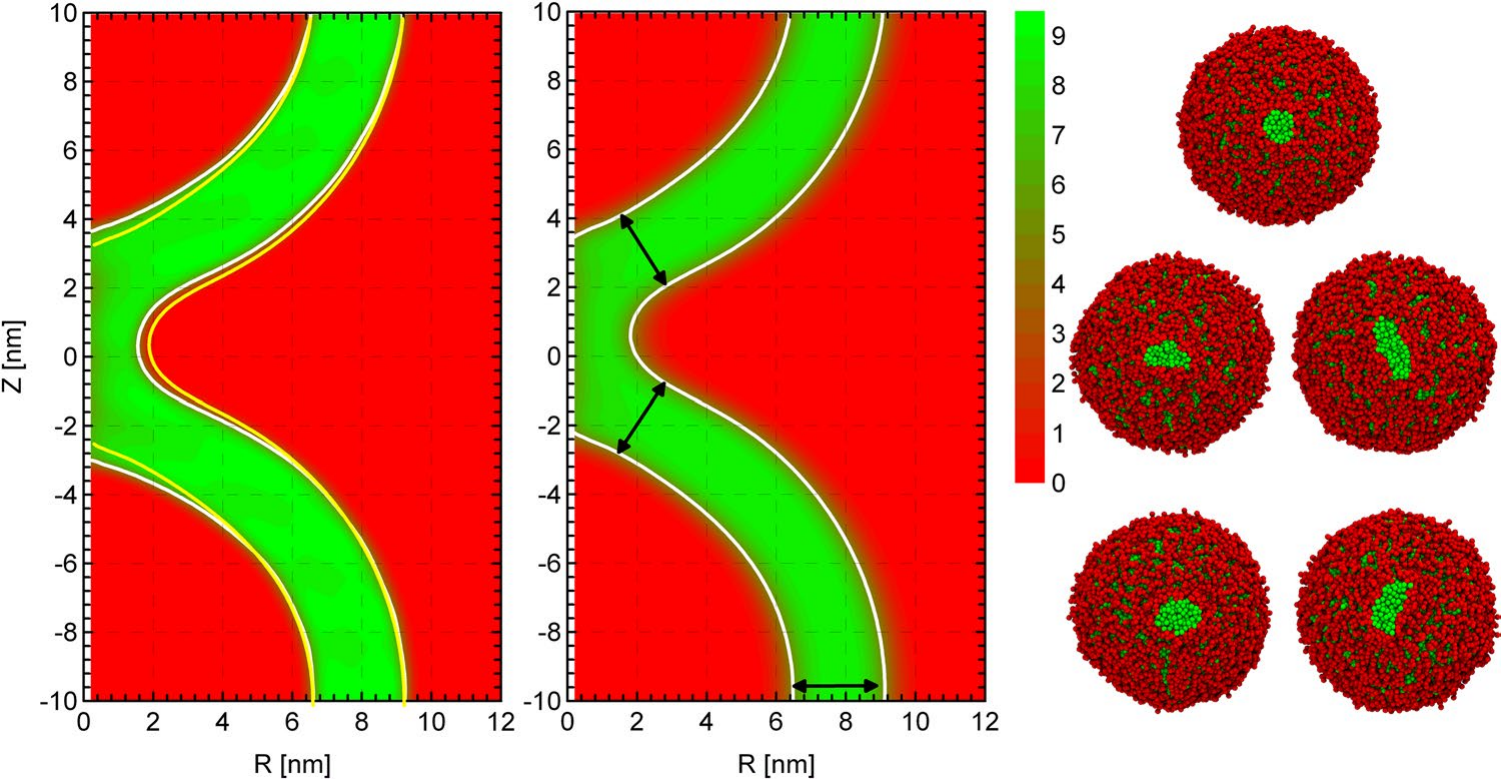
Averaged hydrophobic number density in $\hbox {nm}^{-3}$ of the stalk structure as a function of the axial and radial coordinates. The green color corresponds to the hydrophobic region and the red color to the hydrophilic region. The contour lines depict the interface, in this case corresponding to the half of the maximum density, $5\,\hbox {nm}^{-3}$, but otherwise matching the stalk structure in the external umbrella field in Fig. 2. Left: stalk in the *NVT* ensemble with field-theoretic umbrella potential, Eq. 5; the yellow contour line depicts the interface of the stalk in the unrestrained *NVT* ensemble. Middle: structure of the metastable stalk in the unrestrained *NPT* ensemble. Arrows depict the thinning of the membrane in the vicinity of the stalk center. Right: one representative snapshots of the system with the field-theoretic umbrella potential (top) and four snapshots from unrestrained *NVT* simulations (bottom), showing a cut through the center of the stalk, viewed from the top. Solvent beads are not depicted

Finally, we note that the metastable stalk that we observe in the MFEP calculations is a minimum of the free-energy function, $F(\{m(\mathbf{c})\})$. This situation is similar to self-consistent field theory, where the fluctuations of the molecular conformations are accounted for but the free energy is minimized with respect to fluctuations of the collective density, i.e., the order parameter. Therefore, the shape of the stalk, as specified by the spatial distribution of hydrophobic density, does not exhibit thermal fluctuations and, thus, the stalk adopts a perfectly circular cross-section in the MFEP calculations.

Figure 7 presents two-dimensional density profiles of the metastable stalk between vesicles as a function of the position, *z*, along the stalk axis and the distance, *R*, from the stalk axis. The density has been averaged over the azimuthal angle. Results from a simulation in the *NVT* and *NPT* ensemble are presented. The *NVT* data corresponds to the MFEP calculation, where the field-theoretic umbrella potential has been applied. The results of simulations in the unrestrained *NVT* ensemble are indicated by the yellow contour line. Data in the NPT ensemble are unrestrained. Thus, the order parameter fluctuates and so does the shape of the metastable stalk. Note that these fluctuations result in a broadening of the averaged hydrophilic-hydrophobic interface that is visible (as darker shading) in the middle panel of Fig. 7.

First, we note that the profiles in the unrestrained *NVT* and *NPT* ensemble do not exhibit significant differences, in particular, the diameter of the metastable stalk is essentially the same. This indicates that the use of the *NVT* ensemble along the transformation path does not significantly affect the results, and this observation is in accord with previous studies for apposing, planar membranes (Norizoe et al. 2010; Smirnova et al. 2019).

Second, the arrow in the figure, showing the *NPT* data, indicates that the membrane thickness in the surrounding of the stalk between the vesicles is slightly smaller than the membrane thickness farther away on the vesicle. This thinning is qualitatively comparable to what we have observed for a stalk between two apposing, planar membranes, and is important for the interaction of the stalk with transmembrane anchors of fusion proteins (Smirnova et al. 2019).

Third, Fig. 7 illustrates that the bending of the membranes is less pronounced than in the case of apposing, planar membrane. Qualitatively, the local angle, $\Theta$, between the membranes is $\pi$ in the case of apposing, planar membranes but only of the order $\pi - \varDelta \Theta$ with $\varDelta \Theta \sim d_{\mathrm{st}}/R_\mathrm{v}$ for the vesicles (and $d_{\mathrm{st}} \ll R_\mathrm{v}$). This contributes to the significantly larger (meta)stability of the stalk between vesicles compared to a stalk between two apposing, planar membranes and also partially rationalizes why the stalk is slightly wider for vesicles.

Fourth, we note that the diameter of the metastable stalk, $\langle d_{\mathrm{st}}\rangle _{NVT}\approx 3.6\,\hbox {nm}$, in the unrestrained *NVT* ensemble is somewhat larger than the result from the MFEP calculations, $d_{\mathrm{st}} \approx 3.2\,\hbox {nm}$. The top snapshot in Fig. 7 depicts a snapshot from the MFEP calculations, using the field-theoretic umbrella potential, whereas the four bottom images show cuts through the stalk center, viewed along the stalk axis from unrestrained simulations. Two types of fluctuations contribute to the widening of the average stalk profile. (1) In the case of a perfectly circular stalk, the free energy as a function of the stalk diameter $d_{\mathrm{st}}$ will have a minimum at $d_{\mathrm{st}}$ obtained by the MFEP calculation but we expect that the free energy is not symmetric around this minimum, i.e., radial compression of the stalk is more costly than radial expansion of the stalk—the latter will eventually result in a hemifusion diaphragm in the course of the second stage of fusion. Due to this asymmetry, thermal fluctuations will shift the average stalk diameter, $\langle d_{\mathrm{st}}\rangle _{NVT}$, that is observed in unrestrained simulations, to values larger than $d_{\mathrm{st}}$, at which the free energy adopts its minimum. (2) The snapshots from the unrestrained simulations additionally reveal that there are significant fluctuations in the shape of the stalk cross-section, i.e., the instantaneous shape of the stalk is not perfectly circular but it slightly elongates along a random direction. These shape fluctuations are expected and impart only a small free-energy cost in the vicinity to the spinodal towards the inverted hexagonal phase but tends to increase the estimate of $\langle d_{\mathrm{st}}\rangle _{NVT}$.

## Conclusions

Using molecular dynamics simulations of the coarse-grained MARTINI model in conjunction with the Minimum Free-Energy Path (MFEP) calculations, we have studied the stalk formation between a small vesicle and its periodic image and compared the results to the analog shape transformation between two apposing, planar membranes (Smirnova et al. 2019).

In the zeroth stage of fusion—apposition—a large curvature of the vesicle significantly reduces the dehydration free-energy costs of bringing the vesicles into contact at a given minimal membrane distance, $d_\mathrm{w}$, as quantified by Eq. 8. Moreover, we observe that stalks become metastable fusion intermediates at much larger initial membrane distances, $d_\mathrm{w}$, in the case of vesicles than in the case of apposing, planar membranes, i.e., the distance, $d_\mathrm{w}$, at which fusion may occur is larger for highly curved vesicles than it is for apposing, planar membranes. Both effects contribute to the conclusion that vesicle curvature greatly reduces the dehydration free-energy costs of bringing the membranes to be fused into apposition.

To compare the first stage of fusion—stalk formation—between vesicles and apposing, planar membranes, we have therefore chosen different initial minimal distances, $d_\mathrm{w}=2.3\,\hbox {nm}$ for the vesicle and $d_\mathrm{w}=1.2\,\hbox {nm}$ for the apposing, planar membranes, respectively. For the vesicle system, this $d_\mathrm{w}$ is the largest value, for which the excess free energy, $\varDelta F_{\mathrm{stalk}}$, of the stalk is positive, whereas $d_\mathrm{w}$ for the apposing, planar membranes is the largest value, for which we can still observe a metastable stalk. For these parameters, we observe that the barrier to stalk formation is comparable for the two geometries. This finding agrees with the self-consistent field theory calculations of Lee and Schick (2008). The configurations before the saddle-point reveal pronounced dimples or a narrow pre-stalk. The latter structure consists of a narrow hydrophobic bridge, and the high, concomitant excess free energy originates from a few lipid tails or splayed configurations of the double-tailed lipids that are exposed to the solvent and not completely shielded by the headgroups. Around the barrier, the headgroups begin to shield the hydrophobic tails of the hourglass-shaped bridge from the solvent, and the widening of the stalk lowers the excess free energy, $\varDelta F_s$, along with the reversible transformation path.

The excess free energy, $\varDelta F_{\mathrm{stalk}}$, of the metastable stalk is significantly lower for the vesicle system, and the metastable stalk is wider between vesicles than it is between apposing, planar membranes. This observation qualitatively agrees with previous finding (Lee and Schick 2008; Kawamoto et al. 2015; Risselada et al. 2014) and can be partly rationalized by the reduction of membrane bending as the radius of the vesicle decreases.

We have not studied the second stage of fusion—opening of a fusion pore—but previous self-consistent field calculations (Lee and Schick 2008) and particle-based simulations (Kawamoto et al. 2015) agree that the vesicle curvature reduces the free-energy barriers along this second transformation from a metastable stalk to a fusion pore. Thus, taken together these studies suggest that it is the zeroth stage of fusion that dictates the rate of vesicle fusion and that a high vesicle curvature facilitates fusion because (1) the initial minimal distance, $d_\mathrm{w}$, for fusion to occur and (2) the concomitant hydration free energy decreases with vesicle curvature.

## Footnotes

1 For the system of apposing, planar bilayers at $s=0$, we observe a small perturbation of the shape of *trans* leaflet of the lower membrane in Fig. 2. This deviation from a flat contour is somewhat exaggerated by the choice of the low threshold—hydrophobic density of 2.2 $\hbox {nm}^{-3}$—that defines the membrane interface and presumably arises from the initialization of the path by pulling a single lipid out of the bottom membrane (Smirnova et al. 2010).
